# 

**Published:** 1811-10

**Authors:** 


### NEW FRENCH PUBLICATIONS.

*Cour de Botanique et de Physiologie végétale*, auquel on a joint une description des principaux genres dont les espèces sont cultivées en France, ou qui y sont indigènes; par L. M. Hanin, M. D. &c. in 8vo. 800 pages. Paris 1811.

*Recherches-physico-chimiques*, faites sur la pile; sur la preparation chimique et les propriétés du Potassium et du Sodium; sur la decomposition de l'acide boracique; sur les acides fluorique, muriatique et muriatique oxigéné; sur l'action chimique de la lumiere; sur l'analyse végétale et animale, &c.; par M. M. Gay-Lussac et Thenard, Membres de l'Institut, &c. 2 vol. in 8vo. avec six planches en taille-donce, Paris, 1811.

*Traité de Pharmacie théorique et pratique*, contenant les élémens de l'Histoire naturelle de tous les médicamens, leur préparations chimiques et pharmaceutiques, classées méthodiquement suivant la chimie moderne, avec l'explication des phénomènes, les propriétés, les doses, les usages, les détails relatifs aux arts qui se rapportent à celui de la pharmacie, et à toutes les opérations. On a joint partout les comparaisons des nouveaux poids et mesures, une nouvelle Nomenclature avec les dénominations anciennes, des figures explicatives, et un grand nombre de tableaux; par J. J. Virey, pharmacien en chef à l'hospital militaire de Paris, &c. &c. 2 vol. in 8vo. Paris, 1811.

*Nouvelles remarques sur les hernies abdominales*; par M. Lordat, chef des travaux anatomiques de la faculté de médecine de Montpellier, &c &c. Brochure de 30 pages Montpellier, 1811.

*Nouvelles observations recueillies sur l'éléphantiasis des Arabes*; lues à la Société de l'école de médecine de Paris; par M. Alard. Brochure de 38 pages avec une planche. Paris, 1811.

*Essai de littérature médicale, addressé aux étudians de la faculté de médecine de Strasbourg*; par D. Villars, doyen de la faculté. 8vo. Paris.

*Philosophie médicale, ou vérités fondamentales de la médecine moderne*; par Chortet, ancien médecin militaire de première classe, &c. 8vo. p. 212. Paris.

*Traité d'Hygiène appliquée à la Therapeutique*; par J. B. G. Barbier, M. D. &c. 2 vol. in 8vo. Paris, 1811.

### MONTHLY CATALOGUE OF MEDICAL BOOKS.

A Catalogue of a Modern Collection of Books in Anatomy, Medicine, Surgery, Chemistry, Botany, &c containing the most approved authors for 1811, and 12, to which are added a complete list of the Lectures delivered in London, with their terms, hours of attendance, &c. also Tables of the Pay of the Medical Department of the Army and Navy. A discount of ten per cent allowed for ready money, by J. Callow Medical Bookseller, 10, Crown-court, Princes-street, Soho.

A Paper containing the Results of Eleven Years Practice at the Original Vaccine Pock Institution, No. 44, Broad Street, Golden Square, read at a Meeting of the Governors and Friends of that Establishment, held at Escudier's Hotel, 12th March, 1811, Charles Binney, Esq. Trustee, in the Chair; written by the Medical Board of the Institution.

Essays on the Changes of the Human Body at the different Ages the Diseases to which it is predisposed in each period of life, and the Physiological Principles of its Longevity; the whole illustrated by many Analogies in Plants and Animals. By Thomas Jameson, M. D. 8vo. Longman and Co.

A Collection of Treatises on the Effects of Sol-lunar Influence in Fevers, with an Improved Method of curing them. By Francis Balfour, M. D. First Member of the Medical Board in Bengal. Second Edition, 8vo. Longman and Co.

---

### NOTICES TO CORRESPONDENTS.

Communications have reached us from John Storer, M. D. Robert Beaver, M. D Messrs. E. Harrold, John Ring, W. Hamilton, Observator, and a Norfolk Practitioner.

---

*Printed by E. Hemsted, Great New Street, Fetter Lane.*

